# Keloid Scars: An Updated Review of Combination Therapies

**DOI:** 10.7759/cureus.12999

**Published:** 2021-01-30

**Authors:** Nicholas J Thornton, Benjamin A Garcia, Paige Hoyer, Michael G Wilkerson

**Affiliations:** 1 Dermatology, University of Texas Medical Branch, Galveston, USA

**Keywords:** keloid, keloid scar, keloid treatment, keloid therapy, combination therapy for keloids, combination versus monotherapy for keloids, combination therapy for keloid scars, treating keloids, combination treatment for keloids

## Abstract

Keloid scars are a common yet poorly understood complication of wound healing that can cause a diminished quality of life. Currently, there is little agreement amongst the medical community regarding the best treatment modality for keloids. For this reason, we have created an updated review of the most successful combination therapies for keloid scars and compared their efficacy based on rates of recurrence following treatment. Additionally, these combination therapies have been compared with intralesional triamcinolone acetonide corticosteroid (TAC), which is considered the mainstay monotherapy for keloids. All combination therapies included in our review were shown to produce superior outcomes than TAC monotherapy. We have also found that certain combination therapies are known to produce superior results when used in specific anatomic locations. Intralesional TAC plus intralesional cryotherapy appeared to have the most promising results for non-auricular keloids, and the authors suggest considering this as a first-line treatment. Additionally, the use of surgical excision plus compression therapy achieved superior results for auricular keloids and should be considered first-line for keloids in these locations.

## Introduction and background

Keloid scars are a common yet poorly understood complication of wound healing that can cause a diminished quality of life. The pathogenesis is thought to involve aberrant collagen deposition extending beyond the original wound margins. Many factors have been correlated with a predisposition to keloid formation including specific HLA subtypes, blood type aII, Fitzpatrick skin types V-VI, and age from 10 to 30 years old [1]. Aspects of initial wound management have also been correlated with the formation of keloid scars including delayed debridement, heavy inflammation, and excessive wound tension [1]. Currently, there is little agreement amongst the medical community regarding the best treatment modality for keloids. Recent studies suggest that a multimodal approach is necessary to achieve successful resolution and to decrease rates of recurrence [2-4]. The goal of this review is to compare the most successful current keloid therapies in order to assist physicians in selecting the most appropriate regimen for their patients.

Methods

This review utilized the Medline database via the PubMed search engine to identify reviews published from 2010 to 2020 examining current treatments for keloid scars. Keywords used were “keloid”, “keloid treatment”, “keloid review”, “keloid therapy”. Articles that failed to provide recurrence rates, or a minimum three-month follow up period were excluded. Over 40 articles were identified and nine were selected for inclusion. 

## Review

Results

Due to the subjective nature of comparing aesthetic results, recurrence rate was chosen as the primary metric for comparison. Combination therapies including surgical excision were categorized as "surgical combination therapy". Combination therapies not involving surgical intervention were categorized as "medical combination therapy". 

Mainstay Monotherapy

*Triamcinolone Acetonide Corticosteroid (TAC) Injection. *For years TAC injection has been utilized to treat keloid scars. It is injected in concentrations of 10 to 40 mg/mL administered intralesionally at no less than four- to six-week intervals to avoid adrenal suppression. It has been touted by several studies as the mainstay monotherapy for keloids [1-4]. Its ability to treat keloids is hypothesized to be due to suppression of inflammatory pathways, suppression of fibroblast development and collagen production, and promotion of collagen degradation [1]. TAC is widely available, relatively inexpensive, and easy to administer. When utilized alone as monotherapy, mean recurrence rates are 33% and 50% at one- and five-year follow-up, respectively. The most common side effects of TAC injection are hypopigmentation and dermal atrophy [5].

Surgical Combination Therapies

*Surgical Excision + TAC. *Surgical excision in combination with TAC injections has shown a substantial reduction in recurrence of auricular keloids when compared with either therapy used alone. This therapy is generally accomplished by performing complete excision with either a low-tension primary closure or healing by second intention, plus same-day TAC injections with concentrations ranging from 5-10 mg/mL. Patients then return for monthly TAC injection over the course of four to six months postoperatively. Excision + TAC is shown to have a mean recurrence rate of 15.4% with a follow-up of 12 to 35 months. Primary side effects are hypopigmentation and dermal atrophy [6]. 

*Surgical Excision + Radiation. *Surgical excision with adjuvant radiation therapy performed within 24 hours postoperatively with a total dose no less than 12 Gray has been associated with a mean recurrence rate of 23% at 14 months follow up. This therapy is comparable to surgical excision and TAC combination therapy in terms of efficacy. It has been suggested that surgical excision plus radiation is an acceptable salvage therapy following a failed response to excision plus TAC [7]. Radiation should not be utilized in children, pregnant women, or those with underlying malignancy. The most common side effects are hyperpigmentation and hypopigmentation [4]. The theoretical risk of malignancy as a side effect from radiation therapy has been discussed ever since the implementation of this treatment, however, a 2019 comprehensive review of radiation therapy for keloids suggests that this risk remains negligible [8]. The current consensus regarding radiation therapy for keloids is that it is safe and effective for treating keloid scars with minimal long-term risk.

*Surgical Excision + Pressure Therapy. *Surgical excision followed by pressure therapy is an ideal choice of treatment for keloids located on the ear as this anatomic location is particularly amenable to the implementation of pressure fitting devices such as pressure earrings. This combination is utilized by instructing patients to wear pressure devices over the excised area where the keloid was originally located. The devices often employ the use of magnets to achieve effective compression of the healing area. Pressure therapy is cost-effective and simple but is much less beneficial in anatomic locations outside of the ear. With regards to auricular keloids specifically, this combination therapy is associated with a 6.7%-10.6% recurrence rate at 18-month follow-up period. Because of the exceptional efficacy and low recurrence rate, this combination therapy should always be considered for first-line treatment of keloid scars on the ear [9].

*Surgical Excision + Cryotherapy. *Surgical excision followed by the use of topical cryotherapy has also shown acceptable results for the treatment of auricular keloids. Excision is followed immediately postoperatively by topical cryotherapy using cryoprobes (direct contact method) to attain an impedance value of 1000 KΩ as measured by an impedance meter. Achieving this value generally requires a single freeze-thaw cycle of about 30 seconds [10]. Different size cryo-probes can be selected based on size of the lesion in order to provide an even distribution of contact, and thus even freezing, to the entire keloid. Recurrence rates have been demonstrated as 15% recurrence with a median follow-up of 43 months. The primary side effect following treatment was hypopigmentation, especially in patients with skin of color [10]. Larger keloids located outside of the ear may not benefit as greatly from this combination due to the difficulty in achieving effective freezing on larger anatomic areas.

*Surgical Excision + Mitomycin C (MC). *Surgical excision followed by application of topical MC has recently been shown to produce acceptable results as a combination therapy for the treatment of keloids in various anatomic locations. Mitomycin C is a chemotherapeutic agent that is capable of decreasing fibroblast production and cell division by inhibiting transcription of DNA to RNA. It accomplishes this by creating DNA crosslinks [2]. After excision, gauze soaked with MC at a dose of 1mg/ml is generally applied for 3-5 minutes postoperatively and again at three weeks post-op. Keloid recurrence rates after surgical excision plus MC combination therapy have been shown to average 16.5% with a mean follow-up period of six-month duration [11]. There is potential for dose-dependent adverse effects with MC and it should be carefully dosed at 1mg/ml to avoid these potential complications [11]. Additionally, MC should never be used in pregnant patients due to the potential for harm to the developing fetus [12].

*Surgical Excision + Imiquimod. *Surgical excision followed by application of topical Imiquimod has been shown to have similar but slightly inferior results when compared to excision and MC combination therapy. Imiquimod is a potent immune modulator and functions by stimulating an immune response via synergistic activation of several distinct pathways [13]. Imiquimod stimulates toll-like receptors 7 and 8 leading to activation of nuclear factor-kappa B [13]. Imiquimod also stimulates the expression of tissue necrotic factor-alpha, alpha and gamma interferon, and interleukins 1,6,8, and 12 [13]. Excision plus Imiquimod combination treatment is performed by applying 5% Imiquimod cream to the surgical site nightly for six to eight weeks post excision. Results show a mean recurrence rate of 24.7% with an average follow-up period of six months [11].

Medical Combination Therapies

*TAC + Laser Therapy. *Recently, the use of lasers has shown promising results throughout the field of dermatology. Similarly, combination treatment of keloids with lasers has also shown favorable outcomes. CO2 lasers are thought to work synergistically with TAC by decreasing fibroblast activity thus minimizing production of collagen [14]. The CO2 laser emits light at a wavelength of 10,600 nm that is then absorbed by water. This results in tissue vaporization and effectively creates individual rows of vaporized tissue surrounded by normal skin that are known as Microthermal Zones or MTZ’s [15]. These MTZ’s assist in tissue regeneration by eliciting a robust wound healing response [15]. Studies have analyzed the results of CO2 Laser ablation of keloids followed by monthly intralesional TAC treatment for up to six months following laser procedure. Results have shown a mean recurrence of 15.4% with a mean follow-up of six months. Primary side effects from CO2 laser ablation are pain and hypopigmentation [16].

*TAC + 5-Fluororacil (5-FU). *The use of intralesional 5-FU as a monotherapy to replace TAC has been heavily examined in the recent literature, but most data fails to show superiority to TAC monotherapy [17]. However, TAC+5-FU has been demonstrated to alleviate many of the deleterious side effects associated with TAC monotherapy while retaining slightly improved or similar overall outcomes. Recurrence rates have not been well elucidated for this combination therapy and have ranged between 0% and 47% in various studies [17]. Despite the lack of uniform evidence demonstrating low recurrence, this therapy deems itself worthy of consideration due to its superiority over either of its two ingredients used alone and ease of administration [17]. However, patients may require anesthetic prior to 5-FU injections to improve tolerability.

*TAC + Intralesional Cryotherapy. *TAC has also been combined with intralesional cryotherapy to achieve acceptable results. Weshahy and Abdel Hay et al. demonstrated the effectiveness of intralesional cryotherapy followed by TAC injections over the course of six months. The intralesional cryotherapy is administered by inserting cryo needles at or below the base of the lesion and allowing freezing of the blood supply of the keloid to occur. TAC is subsequently injected into the lesion over the following several months. Results of this study found only a 12% recurrence at six-month follow-up period [18].

Miscellaneous Treatments

A recent meta-analysis by Ellis et al. chose to compare dual therapy to triple therapy for treating keloids and found that there was no overall statistical difference between dual and triple therapy [19]. However, when dual therapy consisting of excision plus radiation was compared to excision, radiation, and another treatment (either hyperbaric oxygen or platelet-rich plasma therapy), a statistical difference was observed [19]. Hyperbaric oxygen and platelet-rich plasma therapy for keloids is not sufficiently studied, but both options have shown promise when used in combination with at least two additional treatment modalities [20,21].

It is worth noting that Interferon therapy has been utilized following surgical excision with acceptable outcomes but was not included in this study due to being published outside of the inclusion timeline. TAC and all included combination therapies are compared as seen in Table 1. 

**Table 1:** Comparison of TAC and combination therapies. This table is inspired by the table created by Ekstein et al. and displays the most effective combination therapies defined as either surgical or medical combination therapy as well as the mainstay monotherapy [2]. Results are compared via recurrence rates and special considerations for each technique are included. TAC: triamcinolone acetonide corticosteroid.

Type	Treatment	Average recurrence rate (%)	Average follow-up (months)	Design	Primary results	Benefits	Authors
Monotherapy	TAC	33%	12 months	Review	TAC injection shows 50-100% regression after treatment but has high recurrence rates	Inexpensive/Easy to administer/Relatively safe	Morelli Coppola et al. [5]
Surgical combination therapy	Surgical Excision + Radiation	23%	14 months	Review	Significant reduction in recurrence when compared to excision or radiation monotherapy	Effective and quick symptom relief/Low recurrence/Fewer treatment sessions required	Mankowski et al. [4]
	Surgical Excision + TAC	15.4%	12-35 months	Review	Significant reduction in recurrence compared to excision or TAC monotherapy	Ideal for auricular keloids/Effective and quick symptom relief/Low recurrence	Shin et al. [6]
	Surgical Excision + Pressure Therapy	10.6%	18 months	Prospective	Excellent results obtained for patients with auricular keloids	Ideal first-line treatment for auricular keloids/Pressure therapy is a minimally invasive, inexpensive adjunct/Lower recurrence rates than Excision + TAC, or Excision + Cryotherapy for auricular keloids	Park et al. [9]
	Surgical Excision + Cryotherapy	15%	43 months	Retrospective	Excellent results obtained for patients with auricular keloids	Ideal for large auricular keloids	Litrowski et al. [10]
	Surgical Excision + Mitomycin C	16.5%	6 months	Review	Similar efficacy to excision + TAC but with potential dose-dependent adverse effects	Less efficacious when compared to excision + TAC/May be useful if TAC not available	Shin et al. [11]
	Surgical Excision + Imiquimod	24.7%	6 months	Review	Higher recurrence compared to excision + MC but with less risk of adverse side effect	Less efficacious when compared to excision + TAC/May be useful if TAC not available	Shin et al. [11]
Medical combination therapy	TAC + Laser Therapy	15%	6 months	Prospective	Superior results than laser or TAC monotherapy	Ideal for large difficult keloids	Garg et al. [16]
	TAC + 5-Fluoruracil	17.5%	3 months	RCT	Superior results and less recurrence compared to TAC monotherapy	Decreased side effects compared to TAC or 5-FU monotherapy	Khalid et al. [17]
	TAC + Intralesional Cryotherapy	12%	6 months	Pilot study	Intralesional cryotherapy performed prior to TAC injection showed superior results, decreased side effects, and a lower recurrence rate than TAC monotherapy	Cost-effective/Useful for most anatomic locations/Widely available	Weshahy et al. [18]

Discussion

TAC injections used as monotherapy for keloids are associated with increased rates of recurrence and greater side effect profile when compared to combination therapy. Even studies that have found no significant difference in resolution or recurrence between TAC monotherapy versus TAC combination therapy have reported a reduction in deleterious side effects when TAC is utilized with additional therapy such as 5-FU [5]. These findings indicate that superior outcomes can be achieved when employing a combined therapy for keloids. Each combination method has unique risks and benefits that must be considered when selecting the most appropriate regimen for the patient.

Patient preferences should be addressed when developing a personalized treatment regimen capable of achieving desired outcomes. Not all patients experience the same keloid symptoms and while some may primarily wish to alleviate pain and pruritus, others are simply concerned with aesthetics. Patients with particularly debilitating symptoms may desire quick resolution despite the invasive nature of procedures. Patients with a history of recurrence or prior history of keloids might benefit from more aggressive initial therapy as well. In contrast, patients who do not experience active side effects and wish to remove keloids solely for cosmetic reasons may prefer a conservative approach that can be performed over the course of several months to minimize discomfort. When treating keloids, a thorough discussion between patient and provider should take place in order to select the combination therapy that is most likely to achieve the best results.

## Conclusions

Intralesional TAC plus intralesional cryotherapy appeared to have the most promising results for non-auricular keloids, and the authors suggest considering this as first-line treatment. Additionally, the use of surgical excision plus compression therapy achieved superior results for auricular keloids and should be considered first-line for keloids in these locations. Comparison of the existing literature on keloid treatment is challenging due to lack of standardization across studies. Many recent studies have variable follow up periods, differences in provider techniques, and widely varying keloid characteristics. This review focused on combination therapy with only two treatment modalities, however, future research may be warranted to assess the benefit of using three or more types of treatment for keloids. Future research with standardization among factors such as keloid characteristics and follow up intervals would be beneficial for the medical community. As our understanding of the pathophysiology of keloid scars continues to improve, we will gain a better appreciation for which characteristics of patients are best suited for each distinct treatment protocol. In the future, effective monotherapy for keloids may be discovered. Until then, current data suggest that a combined approach will yield the most successful results and best patient outcomes.
